# Epidemic Myalgia—Bornholm Disease

**Published:** 1950-04

**Authors:** N. S. Craig

**Affiliations:** Medical Officer, Clifton College


### EPIDEMIC MYALGIA—BORNHOLM DISEASE

BY

N. S. CRAIG, *M.C.*, M.D.

*Medical Officer, Clifton College.*

Daae first described an epidemic of this disease in Norway in 1872. In 1933 Sylvest published an account of a large epidemic on Bornholm, an island in the Baltic.

*Etiology*. The infecting agent is unknown. In 1924 Small reported that he had found a protozoan organism in the erythrocytes of his patients, but other workers have failed to confirm this. Attlee *et al.* suggested that a streptococcus, which they had isolated from the throats of their patients, was responsible. de Rudder in analysing the epidemiological data of poliomyelitis and epidemic myalgia was impressed by the similarity of the two diseases and he suggested that the latter was a variant of poliomyelitis.

The disease chiefly affects young people under thirty years of age. The sexes are equally affected. Pickles, working in the favourable conditions of Wensleydale, considered that the incubation was four days, but others have observed longer periods, e.g. ten to fourteen days. (Crone and Chapman, Rector.) Epidemics usually occur in summer and autumn.

*General Characteristics*. There is an abrupt onset of severe pain usually in the chest or abdomen, but other areas may be affected. Smith (1937) has described a cervical type. The temperature is usually moderately raised though the milder cases may be afebrile. In young children the respiration is rapid (Pickles 1949). Headache is common, but vomiting, cough and diarrhoea are unusual. The symptoms frequently clear up in forty-eight hours, but relapse is very common and some cases take three to four weeks to recover completely.

In the thoracic type a pleural rub has been described in a number of cases (Williamson; Attlee *et al.*). Neurological symptoms have been recorded. Howard *et al.* in an epidemic of 161 cases had five instances of meningitis and three of meningo-encephalitis. McConnell described a case with generalized headache, photophobia and neck rigidity. The C.S.F. contained 20 cells and 60 mgm. of protein.

---

Fifteen cases occurred at Clifton College in the Autumn of 1949. On September 29th a boy of seventeen complained of the sudden onset of pain in the upper abdomen; with each breath it became severe and was associated with pain just below both clavicles. The whole upper abdomen was tender and pressure in this region produced the infra-clavicular pain. He was afrebrile, there was no cough and his appetite was good. The next day he had practically no symptoms, but on the fourth day his temperature rose to 101 deg. and he had severe pain in the right hypochrondrium and right infra-clavicular region. A coarse rub was heard over the lower ribs anteriorly and crepitus could be felt when the right hypochondrium was palpated. The pain rapidly improved, but it was present to some extent for three weeks. On the fourteenth day of the illness an X-ray of the chest showed no abnormality.

On October 1st a boy of eighteen complained of pain in the same distribution as in the first case, and on October 3rd a third similar case was seen. The subsequent cases commenced on the following dates: October 23rd, one; October 24th, two; October 26th, two; October 27th, one; October 28th, three; and November 3rd, three. In five of these cases the pain was entirely abdominal, accentuated by movement and inspiration. There was marked localized tenderness in the lower abdomen. In one case there was nausea, but no other gastro-intestinal symptoms were observed. The pain in these cases lasted five to seven days. During this period a number of boys were seen with less severe pains in the arms, legs and neck. No neurological symptoms were observed.

About this time seven cases were admitted to a Bristol hospital as emergencies: *

Case 1.—Female, seventeen years. Pain in right hypochondrium.

Case 2.—Male, twenty-eight years. Pain right abdomen and shoulder.

Case 3.—Male, eight years. Pain in abdomen and chest.

Case 4.—Female, thirty-eight years. Pain in the right hypochondrium with a friction rub.

Case 5.—Male, thirty-four years. Pain in the righthy pochondrium and shoulder.

Case 6.—Female, ten years. Pain in chest.

Case 7.—Female, forty-three years. Pain in the right chest with a pleural rub.

*Diagnosis.* In cases in which the pain is abdominal diagnosis has to be made from perforated gastric or duodenal ulcer, acute appendicitis and acute cholecystitis. Myalgia rarely produces any gastric or intestinal symptoms other than pain: this is not of an intermittent colicky character. Nausea and vomiting are rare. When the pain is principally in the chest and especially if there is a friction rub, it may be impossible to exclude pleurisy for a few days: pleural effusion has not been reported and there are no moist sounds in the lungs. In older people coronary thrombosis may be suggested by the area and severity of the pain. Treatment is symptomatic.

### REFERENCES

Daae, 1872.

Sylvest, E.—*Epidemic Myalgia: Bornholm Disease*, 1934, Copenhagen and London.

Small, J. C.—*Am. J. M. Sc.*,1924, **168**, 570.

Attlee, W., Amsler, A. M., and Beaumont, D. C.—*Lancet*, 1924, **2**, 492.

de Rudder quoted by McConnell (*q.v.*).

Pickles, W. N.—*Epidemiology in Country Practice*, 1949, John Wright and Sons Ltd. Bristol.

Howard *et al.*—*J.A.M.A.*, 1943, **121**, 925.

McConnell, J.—*Am. J. M. Sc.*, 1945, **209**, 41.

Williamson, B.—*Lancet*, 1924, **2**, 64.

Crone and Chapman; Rector, J. M.—*Am. J. Dis. Child.*, 1935, **1**, 1095.

Smith, R. E.—*Clinical Journal*, 1937, **66**, 330.

* I am indebted to Mr. J. M. Naish for this information.

